# Soluble epoxide hydrolase inhibitors, t-AUCB, downregulated miR-133 in a mouse model of myocardial infarction

**DOI:** 10.1186/s12944-018-0780-y

**Published:** 2018-05-29

**Authors:** Yajun Gui, Da Li, Jingyuan Chen, Yating Wang, Jiahui Hu, Caixiu Liao, Limin Deng, Qunyan Xiang, Tao Yang, Xiao Du, Shilan Zhang, Danyan Xu

**Affiliations:** 1 Department of Cardiovascular Medicine, The Second Xiangya Hospital, Central South University, Changsha, Hunan 410011 China; 2 Department of Geratology, Internal Medicine, the Third Hospital of Changsha, Changsha, Hunan 410011 China; 3 Center for Pulmonary Vascular Disease, FuWai Hospital & Cardiovascular Institute Chinese Academy of Medical Sciences & Peking Union Medical College, Beijing, China; 4 Department of Cardiology, Internal Medicine, Changsha Central Hospital, Changsha, Hunan 410011 China

**Keywords:** Soluble epoxide hydrolase inhibitors, miR-133, Ischemic arrhythmia

## Abstract

**Background:**

It has been demonstrated that soluble epoxide hydrolase inhibitors (sEHIs) are protective against ischemia-induced lethal arrhythmias, but the mechanisms involved are unknown. Previously, we showed that sEHIs might reduce the incidence of ischemic arrhythmias by suppressing microRNA-1 (miR-1) in the myocardium. As miR-1 and miR-133 have the same proarrhythmic effects in the heart, we assumed that the beneficial effects of sEHIs might also relate to the regulation of miR-133.

**Methods:**

A mouse model of myocardial infarction (MI) was established by ligating the coronary artery. The sEHI t-AUCB (trans-4-[4-(3-adamantan-1-yl-ureido)-cyclohexyloxy]-benzoic acid) was administered daily for 7 days before MI. Myocardial infarct size and cardiac function was assessed at 24 h post-MI. The miRNA expression profiles of sham and MI mice treated with or without t-AUCB were determined by microarray and verified by real-time PCR. The incidence of arrhythmias was assessed by in vivo electrophysiologic studies. The mRNA levels of miR-133, its target genes (*KCNQ1* [potassium voltage-gated channel subfamily Q member 1] and *KCNH2* [potassium voltage-gated channel subfamily H member 2]), and serum response factor (*SRF*) were measured by real-time PCR; *KCNQ1*, *KCNH2*, and *SRF* protein levels were assessed by western blotting.

**Results:**

We demonstrated that the treatment with sEHIs could reduce infarct size, improve cardia function, and prevent the development of cardiac arrhythmias in MI mice. The expression levels of 14 miRNAs differed between the sham and MI groups. t-AUCB treatment altered the expression of eight miRNAs: two were upregulated and six were downregulated. Of these, the muscle-specific miR-133 was downregulated in the ischemic myocardium. In line with this, up-regulation of miR-133 and down-regulation of *KCNQ1* and *KCNH2* mRNA/protein were observed in ischemic myocaridum, whereas administration of sEHIs produced an opposite effect. In addition, miR-133 overexpression inhibited expression of the target mRNA, whereas t-AUCB reversed the effects. Furthermore, SRF might participate in the negative regulation of miR-133 by t-AUCB.

**Conclusions:**

In MI mice, sEHI t-AUCB can repress miR-133, consequently stimulating *KCNQ1* and *KCNH2* mRNA and protein expression, suggesting a possible mechanism for its potential therapeutic application in ischemic arrhythmias.

**Electronic supplementary material:**

The online version of this article (10.1186/s12944-018-0780-y) contains supplementary material, which is available to authorized users.

## Background

Life-threatening ischemic arrhythmias occurring following myocardial infarction (MI) are a common cause of sudden cardiac death. Unfortunately, most antiarrhythmic drugs have been challenged in the clinic due to limited effectiveness and proarrhythmic potential [1]. For example, several class III antiarrhythmic drugs prolong the QT interval and increase the risk of potentially lethal torsades de pointes (TdP) arrhythmias in patients with MI [2]. Clearly, there is an unmet clinical need for novel arrhythmia therapeutics.

Epoxyeicosatrienoic acids (EETs) are the main products of arachidonic acid catalysis by the cytochrome P450s. EETs have a wide variety of cardioprotective effects, such as causing marked vasodilation, inhibiting platelet aggregation and adhesion, anti-inflammatory, and modulating lipid metabolism [3–8]. However, most EETs are unstable in vivo and hydrolyzed into the corresponding dihydroxyeicosatrienoic acids (DHETs) by soluble epoxide hydrolase (sEH). sEH inhibitors (sEHIs) can enhance the beneficial effects of EETs by increasing the level of endogenous EETs [9]. Several studies have documented the cardioprotective effects of sEHIs in preventing cardiac arrhythmias both in murine models with cardiac hypertrophy and with MI [10–12]. We previously reported that sEHIs have anti-arrhythmic effects by repressing the activation of nuclear factor κB (NF-κB)–mediated gene transcription in animal models of pressure-overload hypertrophy [10]. However, the exact mechanisms by which sEHIs exert their anti-arrhythmic effect after MI have not been studied.

MicroRNAs (miRNAs) are small noncoding RNAs that negatively interfere with mRNA stability and translation by binding to the 3′ untranslated regions (3′ UTR) of the target mRNAs [13]. Alterations of miRNA expression and function might occur during the pathogenesis of certain heart diseases [14]. For example, miR-133 was enriched in muscle tissues and myogenic cells, and it was found to be involved in diverse physiological processes including carcinogenesis, myocyte differentiation, and disease. More strikingly, the aberrant expression of miR-133 has been linked to many cardiac disorders, such as cardiac hypertrophy, heart failure, myocardial infarction and cardiac arrhythmia [15]. Abnormal miR-133 expression provokes cardiac arrhythmias by repressing several K^+^ channel genes. A study has revealed that miR-133 upregulation increases action potential duration and thereby prolongs the QT interval by decreasing functional expression of the *KCNQ1* (potassium voltage-gated channel subfamily Q member 1)-encoded slow delayed rectifier K^+^ current (I_Ks_) channel in human cardiac progenitor cells [16]. Furthermore, miR-133 can also inhibit the expression of *KCNH2* (potassium voltage-gated channel subfamily H member 2), which encodes the ether-a-go-go related gene (*ERG*) channel subunit responsible for delayed rectifier K^+^ current (I_kr_), resulting in slowed repolarization and prolonged QT interval in the heart [17]. Therefore, miR-133 could be a new target for treating ischemic arrhythmias. We previously demonstrated that sEHIs might reduce the incidence of ischemic arrhythmias by suppressing miR-1 in cardiomyocytes [18, 19]. As miR-1 and miR-133 are clustered on the same chromosome loci and transcribed together in a tissue-specific manner [20], we speculated that miR-133 might also contribute to the anti-arrhythmic action of sEHIs.

The aim of the present study was to complement and extend our earlier studies by investigating whether the beneficial effects of sEHIs are also related to miR-133 expression except miR-1 in a mouse model of MI. To this end, we determined the effects of the sEHI trans-4-[4-(3-adamantan-1-yl-ureido)-cyclohexyloxy]-benzoic acid (t-AUCB) on the expression of miR-133, its target arrhythmia–related genes (*KCNQ1* and *KCNH2*), and serum response factor (*SRF*), an important transcriptional factor in cardiomyocytes.

## Methods

### Surgical procedures and drug administration

All animal protocols were approved by the Animal Use and Care Committee of the Second Xiangya Hospital of Central South University. Male Kunming mice (7 weeks old; Medical Experimental Animal Center, Hunan, China) underwent MI or sham surgery. MI was created by ligating the left anterior descending coronary artery as described previously [21]. The mice were randomly divided into five groups (*n* = 5): (i) Sham, (ii) MI, (iii) 0.001 mg/L t-AUCB+MI, (iv) 0.01 mg/L t-AUCB+MI, (v) 0.1 mg/L t-AUCB+MI. t-AUCB (0.001, 0.1, and 0.1 mg/L) was administered in drinking water for 7 days prior to surgery, and was synthesized in the laboratory of Prof. Bruce D. Hammock (University of California, Davis, CA, USA). The t-AUCB (50 mg) was dissolved in 1 L drinking water followed by 1-h sonication. Then, the stock solution (50 mg/L) was diluted to 0.001, 0.01, and 0.1 mg/L, and was stored at room temperature. Compared with other earlier, such as AUDA, t-AUCB has improved water solubility and better oral bioavailability. Therefore, giving t-AUCB in drinking-water is recommended as a feasible and easy route of administration [22]. The water solubility of t-AUCB is 160 mg/L. Mice were observed to drink approximately 6–7 ml water per day, indicate this procedure gives a dose of approximately 0.2–23 μg t-AUCB per kg per day. There were no significant differences in the daily water intake between each groups. Each mouse was housed in a separate cage in order to monitor the daily water intake.

### Tissue collection

The hearts were removed from the mice 24 h after occlusion. Ventricular tissues within the border zone were dissected to measure the miR-133, *KCNQ1*, *KCNH2*, and SRF levels.

### Infarct size analysis

Frozen ventricles were sliced into 2-mm sections, and the samples were stained with 2,3,5-triphenyltetrazolium chloride (TTC) as previously described [19]. The viable myocardium was stained red, and infarct tissues appeared pale white. The area of infarction and the left ventricle were measured using ImageJ. The infarct size was expressed as a percentage of the total left ventricular area.

### Analysis of cardiac function by echocardiography

Echocardiograms to assess cardiac function were performed at the end of the experiments by using two-dimensional and M-mode measurements as previously described [12].

### In vivo gene transfection

Male Kunming mice were randomly divided into five groups: (i) sham, (ii) MI, (iii) agomir-NC + MI, (iv) agomir-133 + MI, (v) agomir-133 + 0.1 mg/L t-AUCB+MI. In a preliminary study, we tested different doses of agomir (10,25,40 nM) in the animal model,and we found that the agomir dose (25 nM) were able to increase miR-1 level in MI mice for above 15-fold compared with the control (Additional file 1: Figure S1). Therefore, agomir-133 25 nM was chosed to be utilized in subsequent annimal experiments. Agomir of miR-133 (25 nM of ribonucleotide diluted in 0.2 mL saline) were injected via the tail vein after occlusion. As a control, agomir-negative control (agomir-NC) were injected via the tail vein. Experimental measurements were made 24 h after tail vein injection.

### In vivo electrophysiologic studies in mice

In vivo electrophysiologic were performed as previously described [10]. Standard pacing protocols were used. Each animal underwent an identical pacing and programmed stimulation protocol.

### Real-time PCR and miRNA expression

For mRNA, 1 μg RNA was reverse-transcribed using a RevertAid First Strand cDNA Synthesis Kit (Fermentas). For miRNA, 200 ng total RNA was reverse-transcribed using a TaqMan MicroRNA reverse transcription (RT) Kit (Applied Biosystems). Quantitative PCR (qPCR) was performed with SYBR Premix Ex Taq (TaKaRa) and TaqMan Universal Master Mix II (Applied Biosystems) for mRNA and miRNA, respectively.

### Western blotting

Western blotting was performed as described previously [23] with primary anti-bodies against KCNQ1 (1:300, rabbit monoclonal; Abcam), KCNH2 (1:1000, rabbit monoclonal; Sigma), and SRF (1:1000, rabbit monoclonal; Cell Signaling Technology). β-Actin (1:1000, rabbit monoclonal; Abcam) was used as the internal control. Bands were quantified using ImageJ.

### Data analysis

All data are expressed as the mean ± SEM and were analyzed using GraphPad Prism 5.0 and SPSS 21.0 software. Groups were compared by one-way analysis of variance (ANOVA), followed by Bonferroni’s multiple comparison test. *P* < 0.05 was considered statistically significant.

## Results

### Effect of t-AUCB on infarct size

**Fig. 1:**
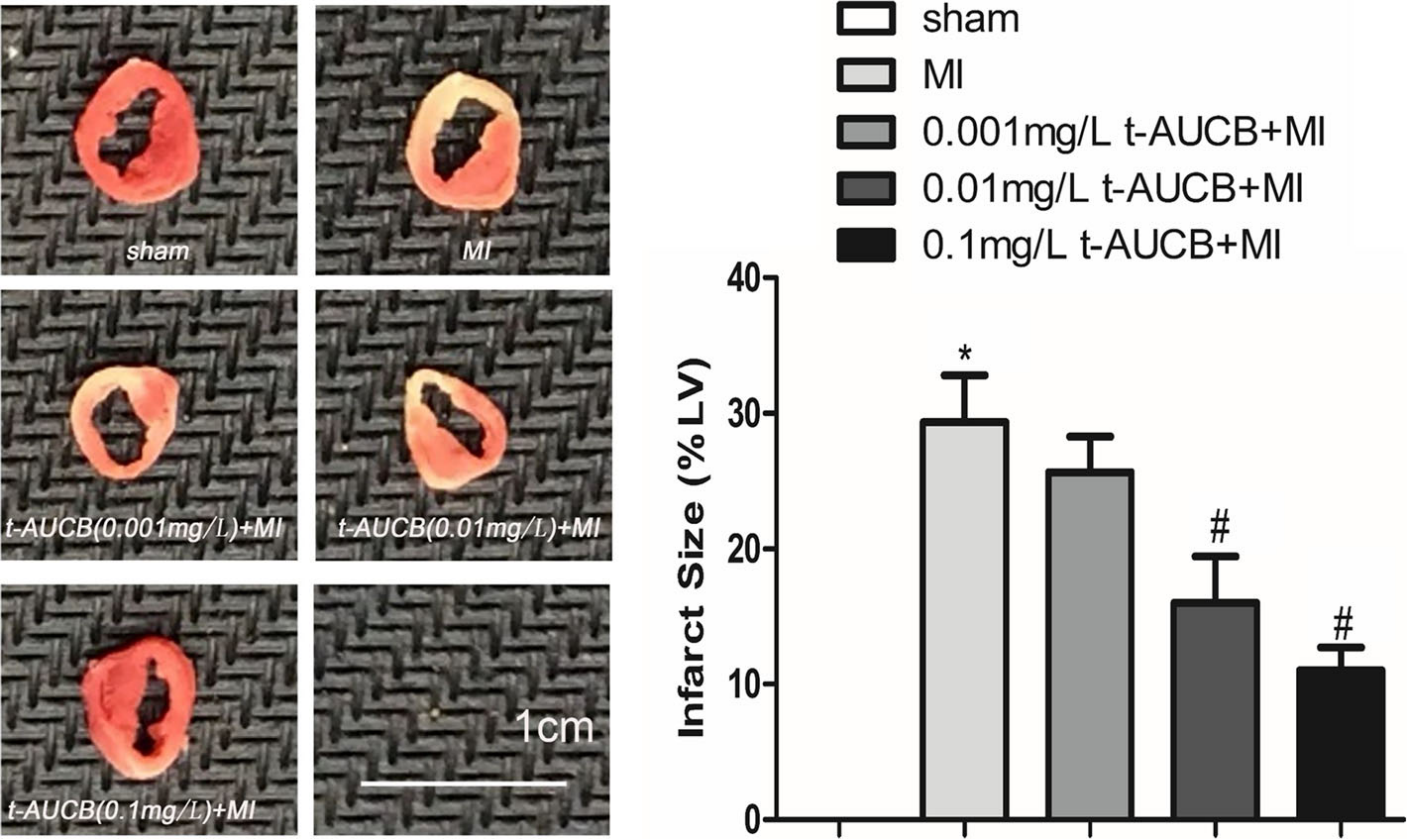
t-AUCB decreased infarct size in MI mice. Representative images of 2,3,5-triphenyltetrazolium chloride (TTC) staining in t-AUCB-treated or control hearts (left). Surviving tissue stained red with TTC and infarcted tissue was white. Infarct size expressed as percentage of left ventricular area for each group (right). Bars represented mean ± SEM; *P<0.05 vs. sham group; #*P*<0.05 vs. MI group. *n* = 3

Figure 1 presents the results. Compared with the untreated MI group, the myocardium infarct size was reduced from 30 to 26%, 16, and 11% in MI mice treated with 0.001 mg/L, 0.01 mg/L, and 0.1 mg/L t-AUCB, respectively (all *P* < 0.05).

### Assessment of left ventricular function by echocardiography

**Fig. 2:**
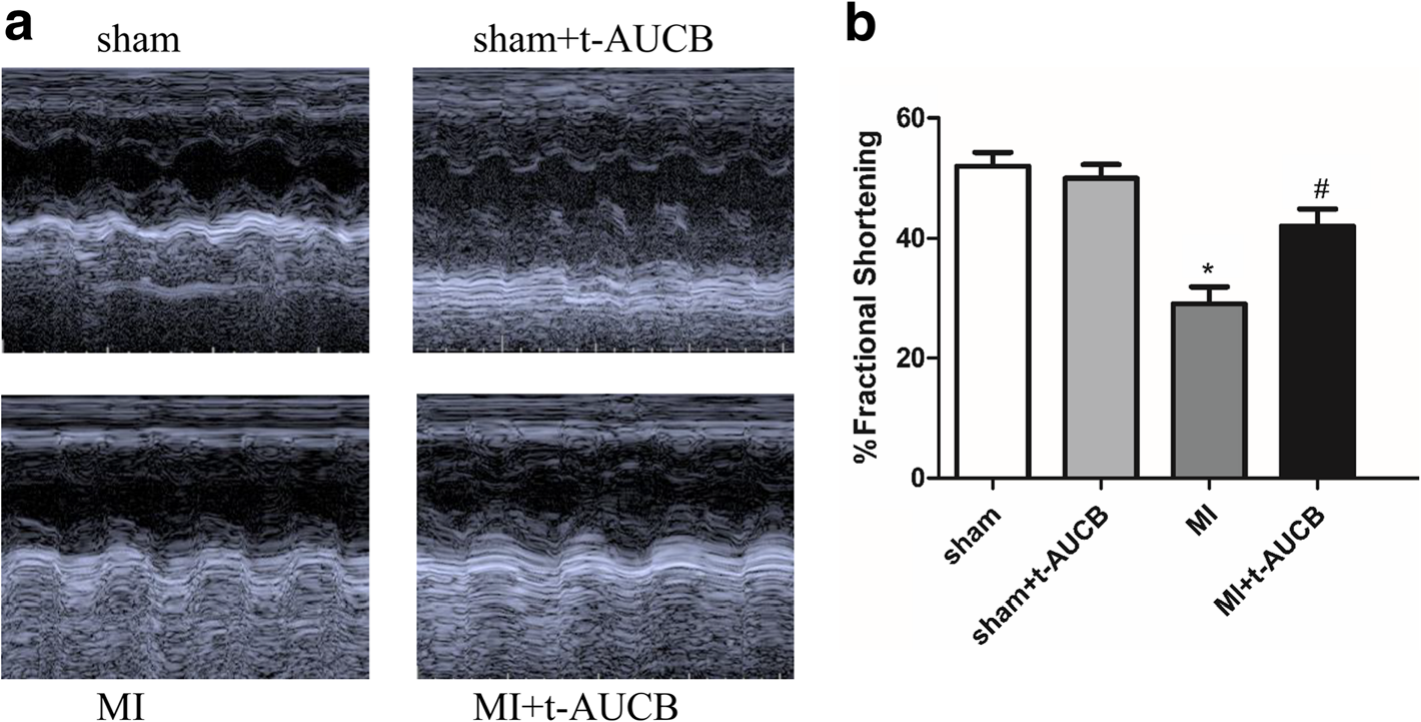
Assessment of cardiac function. **a** Examples of M-mode echocardiography in sham-operated, MI, sham and MI treated with t-AUCB after 1 week of treatment, showing evidence of cardiac failure with chamber dilatation in MI mice. T-AUCB prevented the development of chamber dilatation in MI mice. Summary data are shown in b. **b** Fractional shortening (FS), a surrogate of systolic function, was calculated from left ventricule dimension as follows: FS = ((EDD-ESD)/EDD) × 100%. Data were expressed as mean ± SEM; EDD, end diastolic dimension; ESD, end systolic dimension; *P<0.05 vs. sham group; #*P*<0.05 vs. MI group. *n* = 5

We assessed the chamber size and systolic function in sham and MI mice treated with or without t-AUCB using echocardiography. As shown in Fig. 2a using M model echocardiography, compared with untreated MI mice, mice treatedt with t-AUCB resulted in a significant improvement in left ventricular systolic function. Figure 2b summarized the percentage of fractional shortening from sham and MI mice treated with or without t-AUCB. There were no beneficial effects were observed in sham-operated mice after one-week treatment with t-AUCB or without.

### miRNA expression profiles of ischemic myocardium from sham and MI mice treated with or without t-AUCB

We performed microarray to analyze the miRNA profile changes between sham and MI mice treated with or without t-AUCB (Additional file 1: Figure S2A). The expression levels of 14 miRNAs differed between the sham and MI groups: 10 were upregulated and four were downregulated (fold change ≥2,Additional file 1: Table S1 and Table S2). The expression of eight miRNAs in the t-AUCB–treated MI mice was altered as compared with the untreated MI mice: two were upregulated and six were downregulated. Among them, two proarrhythmic miRNAs, i.e., miR-1 and miR-133, were downregulated in the MI mice after t-AUCB treatment (Additional file 1: Figure S2B). As we have previously demonstrated the role of miR-1 in the ischemic arrhythmia–related gene network [18, 19], we wanted to explore the regulatory function of miR-133 in arrhythmia in the present study. To confirm the microarray results, the changes in miR-133 expression were validated using qRT-PCR. miR-133 expression was increased by 3.1-fold in the MI group as compared with the sham group. However, miR-133 levels were decreased to 46.7% in MI mice treated with 0.1 mg/L t-AUCB compared with untreated MI mice (Additional file 1: Figure S2C, *P* < 0.05). However, t-AUCB showed no effect on the expression of miR-133 in sham-operated mice.

### Effects of t-AUCB on primary neonatal mouse ventricular myocytes viability

To determine whether t-AUCB had an influence on cell viability, neonatal mouse ventricular myocytes were exposed for 3 h to wide range of t-AUCB concentrations (0, 0.1,0.5,1,10,20,35,50,100 μM). After 24 h, the cell viability was determined using LDH assay. Our results showed that t-AUCB concentration ranging from 20 to 100 μM significantly decreased the cell viability (Additional file 1: Figure S3A). Based on this findings, t-AUCB 20 μM was selected to be utilized in subsequent in vitro experiments in neonatal mouse ventricular myocytes. The miR-133 activator agomir-133 was transfected into cells to construct the miR-133 overexpression model. The agomir-133 (200 nM) alone or combination with t-AUCB 20 μm did not significantly affect the cell viability (Additional file 1: Figure S3B). The results suggested that t-AUCB (20 μM) and agomir-133 had no effect on cell viability. Consistently, our previous study demonstrated that t-AUCB and agomr-133 also had no effect on cell viability by using MTT assay [18].

### Effects of t-AUCB on arrhythmias in MI mice transfected with agomir-133

We next performed in vivo electrophysiologic studies (EPS) to test whether sEHIs have salutary effects on ischemic arrhythmias in t-AUCB treated and untreated MI mice at one week as previously described [19]. At baseline, 7 of 10 MI mice (70%) had inducible ventricular tachycardia (VT) during programmed stimulation. Compared with the MI group, the incidence of VT decreased to 50% in MI mice treated with 0.1 mg/L (*P* < 0.05). The susceptibility to increased ventricular arrhythmias was significantly suppressed in MI mice treated with sEHIs. In contrast, transfection of miR-133 agomir promoted ischemic arrhythmias. However, co-application of 0.1 mg/L t-AUCB and miR-133 agomir could rescue this effect. No spontaneous arrhythmias were observed in sham-operated mice treated with and without t-AUCB. Summary data were shown in Table 1.

**Table 1:** t-AUCB protected against ischemic arrhythmia inducibility in MI mice transfected with agomir-133

Groups	VT	AF
sham(*n* = 6)	0	0
MI(*n* = 10)	7 (70%)*	2 (20%)
t-AUCB(0.1 mg/L) + sham(*n* = 6)	0	0
t-AUCB(0.1 mg/L) + MI(*n* = 10)	5 (50%)^#^	1(10%)
agomir-NC + MI(*n* = 6)	4 (67%)	1(17%)
agomir-133 + MI(*n* = 10)	9 (90%)^#^	1(10%)
agomir-133 + t-AUCB(0.1 mg/L) + MI(*n* = 5)	4 (80%)^&^	1 (20%)

Data represent mean ± SEM

Before the MI surgery or sham-operated, mice were randomized to receive either drinkingwater or t-AUCB (0.1 mg/L) for 1 week. Mice were transfected with agomir-NC or agomir133 (25 nM) via the tail vein after occlusion. Measurements were made 24 h after MI. Results in the table were incidence of inducible ventricular tachycardia

*VT* ventricular tachycardia, *AF* atrial fibrillation

^*^P<0.05 vs. sham group

^#^*P* < 0.05 vs. MI group

^&^*P*<0.05 vs agomir-133 + MI group, *n* = 5–10 for each group

### Effects of t-AUCB on miR-133, KCNQ1 and KCNH2 mRNA levels in MI mice

**Fig. 3:**
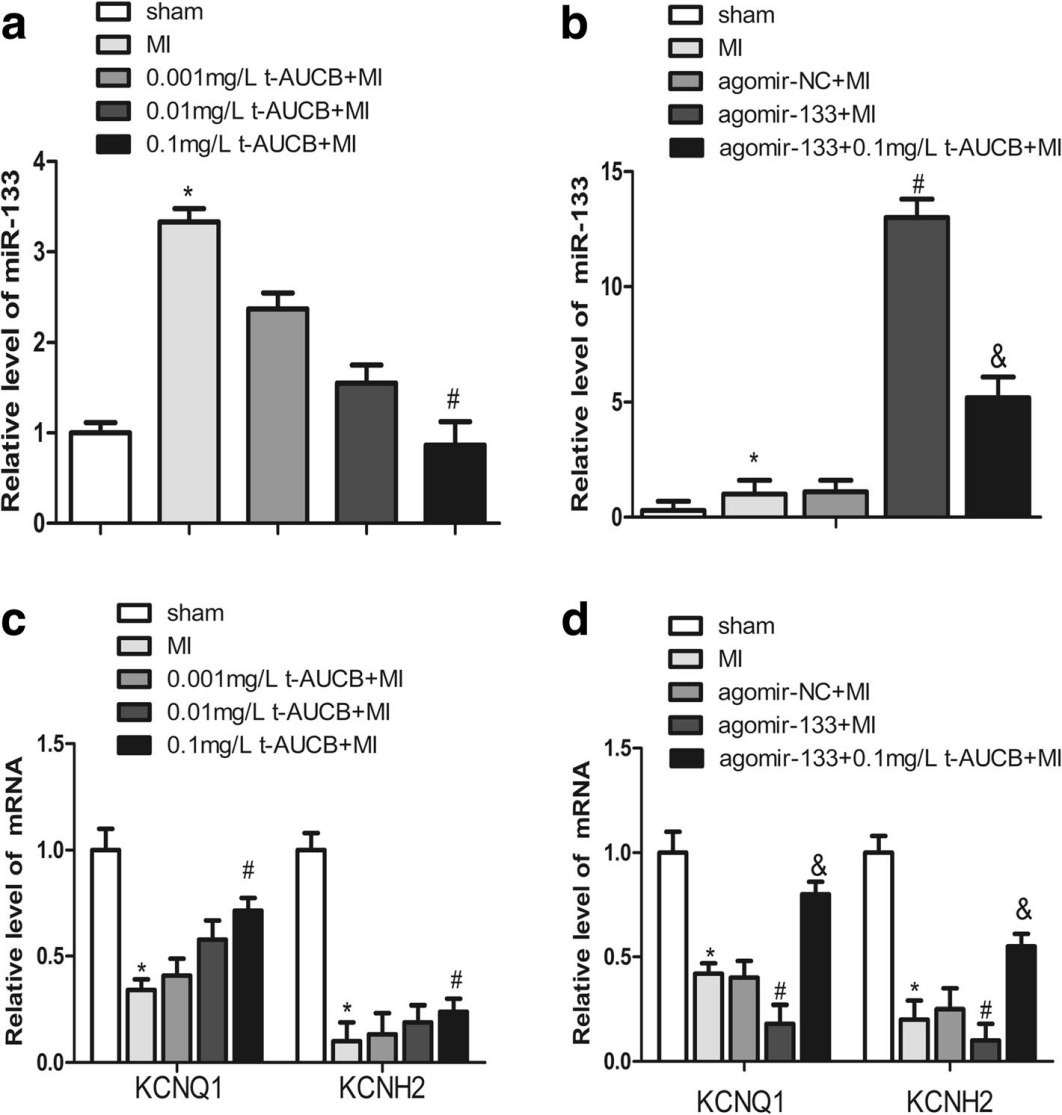
t-AUCB prevented upregulation of miR-133 and restored the expression of *KCNQ1* and *KCNH2* mRNA in ischemic myocardium. **a** Ischemic upregulated miR-133 expression in MI hearts, while t-AUCB suppressed miR-133 expression in a dose-dependent manner. miR-133 level were quantificated by real-time PCR with RNA samples isolated from mice hearts 24 h after MI. **b** The upregulation of miR-133 was exacerbated by agomir in MI hearts, but alleviated by t-AUCB. **c** Ischemic downregulated *KCNQ1* and *KCNH2* mRNA expression in MI hearts, while t-AUCB restored *KCNQ1* and *KCNH2* mRNA expression in a dose-dependent manner. **d** Levels of both *KCNQ1* and *KCNH2* mRNA expression were reduced in MI and the reduction was exacerbated by agomir-133, but alleviated by t-AUCB. Data were expressed as mean ± SEM; *P<0.05 vs. sham group; #*P*<0.05 vs. MI group; &*P*<0.05 vs agomir-133 + MI group, *n* = 5

miR-133 plays an important role in ischemic arrhythmogenesis. Here, we determined the effects of t-AUCB on miR-133 expression in the ischemic myocardium of MI mice. miR-133 expression was increased by 3.3-fold in the MI group as compared with the sham group (Fig. 3a, *P* < 0.05). The upregulated miR-133 was abrogated in a dose-dependent manner in MI mice treated with t-AUCB (Fig. 3a). Compared with the untreated MI group, miR-133 levels were decreased to 70, 47, and 27% in the MI mice treated with 0.001, 0.01, and 0.1 mg/L t-AUCB, respectively (*n* = 5, all *P* < 0.05).

We injection the agonist miR-133 agomir (25 nM) via the tail vein and found that agomir treatment caused a 13.0-d increase in miR-133 level in the MI mice (Fig. 3b, *P* < 0.05). This increased tendency of miR-133 was abolished by pretreatment with t-AUCB. miR-133 level were decreased to 40% in the agomir-133 + 0.1 mg/L t-AUCB+MI group as compared to the agomir-133 + MI group (Fig. 3b, *P* < 0.05).

We used computational predictions to identify the possible miR-133 targets. TargetScan indicated that some arrhythmia-related mRNA encoding K channels, such as *KCNQ1* and *KCNH*2, as possible targets of miR-133. As miRNAs can affect the stability of specific target mRNAs through post-transcriptional repression, we investigated the effects of miR-133 on the expression of *KCNQ1* and *KCNH2* mRNA. *KCNQ1* and *KCNH2* mRNA levels were decreased to 34 and 10%, respectively, in the ischemic myocardium of the MI group as compared with the sham group (Fig. 3c, all *P* < 0.05). t-AUCB upregulated *KCNQ1* and *KCNH2* mRNA expression dose-dependently(Fig. 3c, all *P* < 0.05). Compared with the untreated MI group, *KCNQ1* mRNA expression was increased 1.2-fold, 1.7-fold, and 2.1-fold in MI mice treated with 0.001, 0.01, and 0.1 mg/L t-AUCB, respectively. Significant differences were only found for the 0.1 mg/L t-AUCB+MI group. Likewise, *KCNH2* mRNA expression was increased 1.33-fold, 1.9-fold, and 2.4-fold in MI mice treated with 0.001, 0.01, and 0.1 mg/L t-AUCB, respectively. A significant increase in *KCNH2* mRNA expression was found in the 0.1 mg/L t-AUCB+MI group, but not in other groups.

We used the agomir to further investigate the link between miR-133, *KCNQ1* and *KCNH2* mRNA, and t-AUCB. *KCNQ1* and *KCNH2* mRNA level were significantly decreased in the hearts of MI mice as compared with the sham mice (all *P* < 0.05). *KCNQ1* and *KCNH2* mRNA level were decreased to 43 and 50%, respectively, in the agomir-133 + MI group as compared to the MI group (Fig. 3d, all *P* < 0.05). This reduction was reversed by the 0.1 mg/L t-AUCB pretreatment, which caused a 4.4-fold and 5.5-fold increase in *KCNQ1* and *KCNH2* mRNA expression, respectively (Fig. 3d, all *P* < 0.05).

### Effects of t-AUCB on KCNQ1 and KCNH2 protein in MI mice

**Fig. 4:**
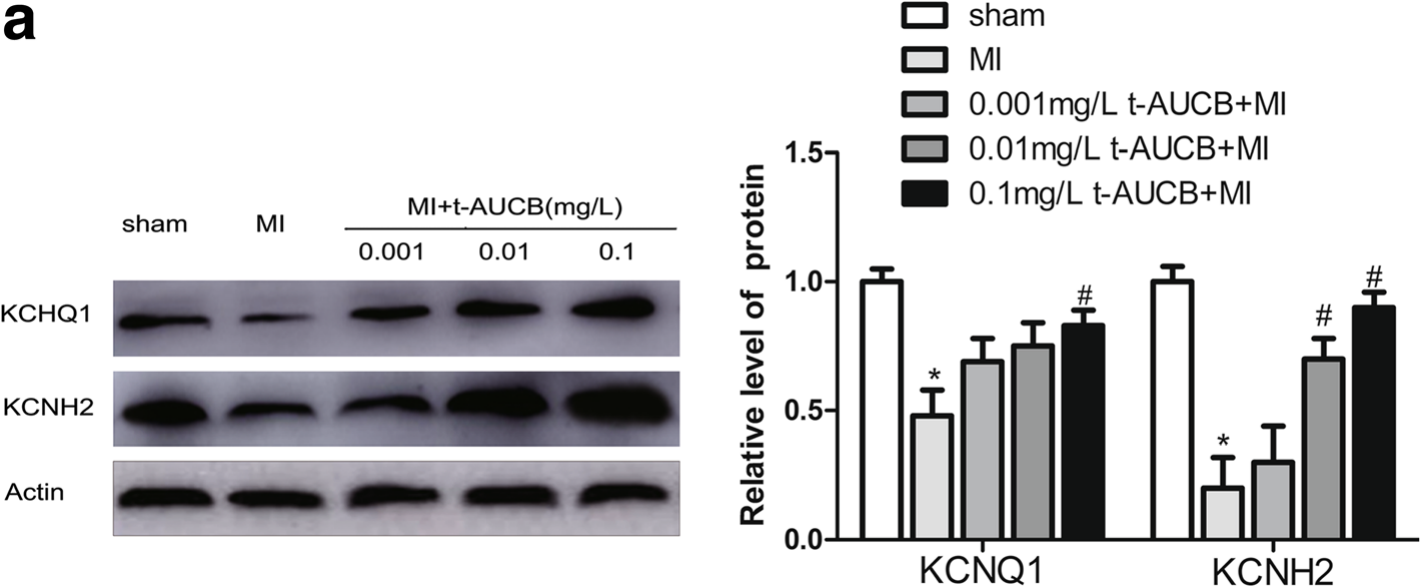
t-AUCB restored the expression of *KCNQ1* and *KCNH2* at the protein level in ischemic myocardium. **a** Ischemic downregulated *KCNQ1* and *KCNH2* protein expression in MI hearts, while t-AUCB restored *KCNQ1* and *KCNH2* protein expression in a dose-dependent manner. Measurements were made 24 h after MI. Left, examples of western blot bands; Right, quantitation as mean ± SEM. *P<0.05 vs. sham group; #*P*<0.05 vs. MI group; *n* = 5

Western blotting revealed that compared with the sham group, *KCNQ1* and *KCNH2* protein levels were decreased to 48 and 20%, respectively, in the ischemic myocardium of the MI group (Fig. 4; all *P* < 0.05); t-AUCB reversed these changes. Compared with the untreated MI group, *KCNQ1* protein expression was increased by 1.44-fold, 1.56-fold, and 1.72-fold in MI mice treated with 0.001, 0.01, and 0.1 mg/L t-AUCB, respectively. Significant differences were found for the 0.1 mg/L t-AUCB+MI group. Likewise, *KCNH2* protein expression was increased by 1.5-fold, 3.5-fold, and 4.5-fold in MI mice treated with 0.001, 0.01, and 0.1 mg/L t-AUCB, respectively. However, there was no significant difference in the 0.001 mg/L t-AUCB+MI group.

### Potential role of SRF in miR-133 reduction by t-AUCB

**Fig. 5:**
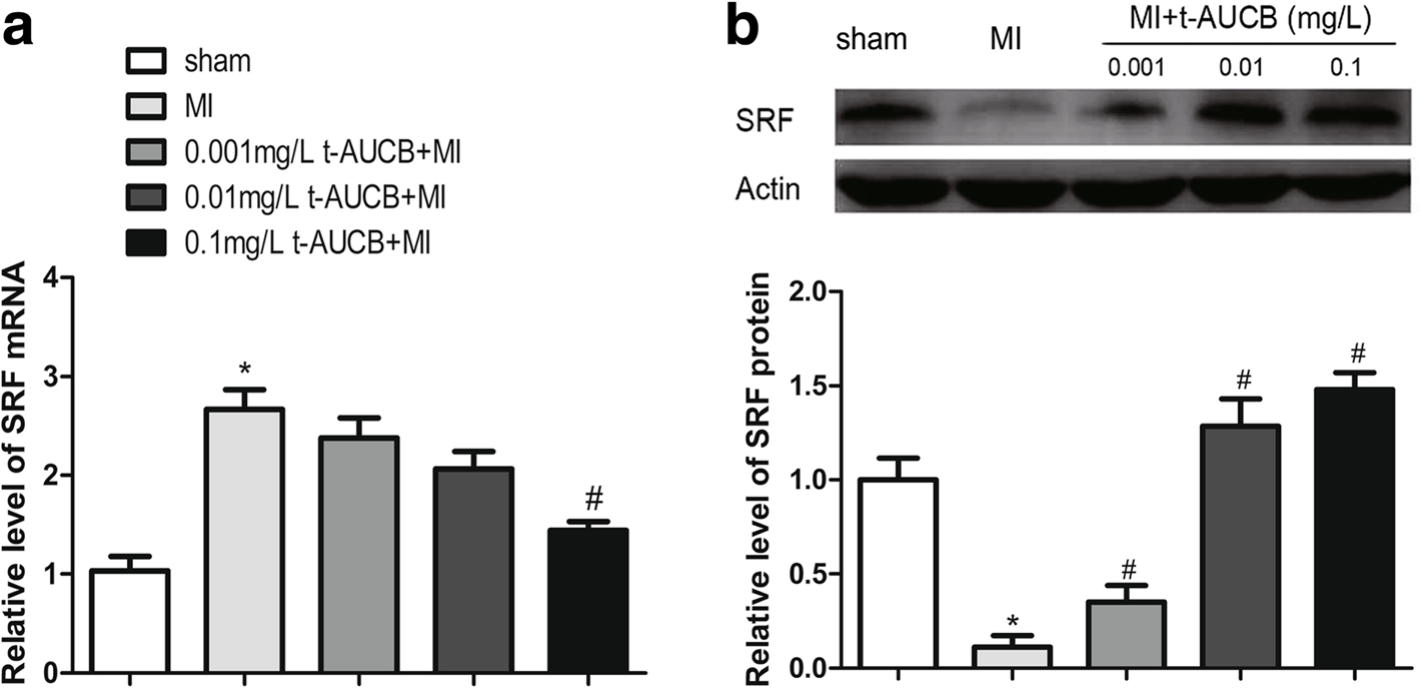
SRF signaling pathway participated in regulation of miR-133 by sEHi. **a** Ischemic upregulated SRF mRNA expression in MI hearts, while t-AUCB suppressed SRF mRNA expression in a dose-dependent manner. SRF mRNA level were quantificated by real-time PCR with RNA samples isolated from mice hearts 24 h after MI. **b** Ischemic downregulated SRF protein expression in MI hearts, while t-AUCB restored SRF protein expression in a dose-dependent manner. Measurements were made 24 h after MI. Top, examples of western blot bands; Bottom, relative expression level of SRF protein. Quantitation as mean ± SEM. *P<0.05 vs. sham group; #*P*<0.05 vs. MI group; *n* = 5

*SRF* is a well-known important transcription factor in the cardiovascular system, and plays an important role regulating miRNA biogenesis [24] the present study, we analyzed *SRF* expression in MI mice pretreated with t-AUCB for 7 days. *SRF* mRNA levels in the ischemic myocardium of the MI group were increased by 2.7-fold as compared with the sham group (Fig. 5a, *P* < 0.05).

The upregulated *SRF* mRNA expression was abrogated in a dose-dependent manner in MI mice treated with t-AUCB (Fig. 5a, *P* < 0.05). Compared with the untreated MI group, miR-133 levels were decreased to 90, 74, and 53% in the MI mice treated with 0.001, 0.01, and 0.1 mg/L t-AUCB, respectively. Significant differences were only found for the 0.1 mg/L t-AUCB+MI group.

There was an inverse correlation between *SRF* mRNA and SRF protein expression. Unlike the expression of *SRF* mRNA, SRF protein was decreased to 11% in MI mice as compared with sham mice (Fig. 5b, *P* < 0.05); t-AUCB pretreatment reversed this reduction. Compared with the untreated MI group, SRF protein expression was increased by 2.9-fold, 12.3-fold, and 14.0-fold in MI mice treated with 0.001 mg/L, 0.01 mg/L, and 0.1 mg/L t-AUCB, respectively (Fig. 5b, all *P* < 0.05).

## Discussion

Our study yields several novel findings. First, the proarrhythmic factor miR-133 is upregulated in response to MI, and the sEHI t-AUCB negatively regulates miR-133 expression. Second, we demonstrate, for the first time, that t-AUCB can abolish the repressing effects of miR-133 on *KCNQ1* and *KCNH2* mRNA and protein in MI mouse hearts. Finally, the activator *SRF* might trigger the t-AUCB–induced miR-133 downregulation. These findings not only aid understanding of the mechanisms underlying the anti-arrhythmic effects of sEHI, but also advance the idea of miRNAs that might serve as potential drug targets.

Recently, sEHIs were been found to be effective in ischemic arrhythmia [10–12]. Previously, we reported that sEHIs can reduce the incidence of ventricular arrhythmias in mouse models with cardiac hypertrophy [10]. Similarly, Shrestha et al. [11] reported that t-AUCB could significantly prevent electrocardiographic (ECG) abnormalities, such as prolongation of the QTc interval and pathological Q-wave formation in isoproterenol-induced MI rats. However, there have been few experimental studies to clarify the mechanisms of the anti-arrhythmic effects of sEHIs. In previous studies, we reported that the sEHI-induced cardioprotective effect was mediated in part by restoring the impaired *KCNJ2* (potassium voltage-gated channel subfamily J member 2)/Kir2.1 and *GJA1* (gap junction protein alpha 1)/Cx43 mRNA and protein expression in cardiomyocytes by suppressing miR-1, which might be triggered by PI3K (phosphatidylinositol 3-kinase)/AKT pathway activation [18, 19]. miR-1 and miR-133 have the same effects on cardiac arrhythmia, as they are both proarrhythmic [17, 25]. Therefore, we hypothesize in the present study that the beneficial effects of sEHIs might also be related to the regulation of miR-133.

Here, we observed the upregulation of miR-133 in ischemic myocardium at 24 h post-MI compared with sham group by using miRNA microarray in a mouse model of MI. The change was confirmed by real-time PCR. In contrast, Kuwabara et al. [26] reported that miR-133 expression was decreased in the border zone at 24 h after coronary ligation in a mouse model of MI; in situ hybridization determined that the hybridization signal of miR-133 had almost disappeared. This could be explained by the fact that surviving cardiomyocytes can release miR-133a–containing exosomes into the circulating blood after calcium ionophore stimulation, resulting in decreased miR-133 expression in the border zone of the infarcted myocardium and elevated levels of circulating miR-133. Similarly, Zile et al. [27] reported that patients with acute MI had significantly increased serum levels of miR-133a and that it could be used as a biomarker of cardiomyocyte death. The elevated plasma miR-133 was believed to mainly originate from the infarcted myocardium and the border zone.

Here, we demonstrate for the first time that the sEHI t-AUCB dose-dependently suppresses miR-133 upregulation in the ischemic myocardium, which might be responsible for the anti-arrhythmic effect of the sEHI. Many studies have shown that miR-133 upregulation might be a proarrhythmic factor in the heart. Shan et al. [17] reported that increased miR-133 expression contributed to arsenic-induced cardiac electrophysiological disorders by repressing ERG protein levels in a guinea pig model. miR-133 knockdown by the antisense molecule AMO-133 prevented QT prolongation and QRS widening by restoring ERG protein expression. Moreover, Belevych et al. [25] reported that miR-133 upregulation led to abnormal myocyte Ca^2+^ handling and increased propensity for arrhythmogenesis. Therefore, sEHI downregulation of miR-133 might confer protection against arrhythmia.

In agreement with its miR-133–reducing effect, we demonstrate that t-AUCB restored the expression of the miR-133 target genes, i.e., *KCNQ1* and *KCNH2* mRNA and protein, in the ischemic myocardium. *KCNQ1* and *KCNH2* mRNA and protein expression were decreased in the ischemic myocardium, whereas t-AUCB restored their expression in a dose-dependent manner, suggesting a dose–effect relationship between sEHIs and *KCNQ1* and *KCNH2* mRNA and protein. *KCNQ1* and *KCNH2* mRNA and protein are directly negatively modulated by miR-133, which can target the 3′ UTR of *KCNQ1* and *KCNH2* mRNA [14, 28, 29].

More important, we further demonstrated that sEHi t-AUCB could restore the expression of *KCNQ1* and *KCNH2* mRNA, which were repressed by the agonist miR-133 agomir. The result further demonstrated that sEHi indirect effected the expression of *KCNQ1* and *KCNH2* mRNA via suppression miR-133. Therefore, we speculated that a sEHI would affect *KCNQ1* and *KCNH2* mRNA and protein expression, in part by suppressing miR-133. However, the mechanism responsible for miR-133 downregulation by t-AUCB remains poorly understood.

SRF, an important transcription factor, regulates numerous genes involved in cell proliferation and differentiation. In our study, SRF protein was downregulated in the ischemic myocardium at 24 h post-MI. In contrast, Lu et al. [30] and Shan et al. [31] reported that SRF protein levels were increased by 1.6-fold and 1.4-fold in rat hearts at 12 h and 3 months, respectively, after MI. The divergent results reflect differences in research models or sampling times. Few experimental studies have clarified the effect of ischemic stimulation on *SRF* mRNA expression. In the present study, *SRF* mRNA levels were increased by 2.7-fold in the ischemic myocardium. These findings indicate that SRF expression is regulated at both transcriptional and post-transcriptional level in MI mouse hearts.

Our results showed opposite expression patterns for *SRF* mRNA and protein. That protein synthesis was disproportionate to the mRNA levels could be explained as follows: 1) gene expression involves two main stages, namely transcription and translation. in eukaryotic cells, transcription and translation are spatially and temporally separate; 2) gene expression is regulated at multiple levels, e.g., transcriptional, post-transcriptional, translation, and post-translational modification; 3) the opposite expression patterns of *SRF* mRNA and protein might be due to the difference in sampling time. mRNA expression might peak while protein production is still increasing, or protein is being synthesized while mRNA is being degraded [32].

It has been established that SRF positively regulates miR-1 expression in the heart [15]. By contrast, it has been proposed that SRF suppresses miR-133 expression [15, 20, 24, 33]. Consistently, the present study demonstrates an inverse relationship between SRF protein and miR-133 expression. We found that the SRF protein downregulation was accompanied by increased miR-133 levels after MI. In line with this, Angelini et al. [34] reported that miR-133 was downregulated in transgenic mice with cardiac-specific overexpression of SRF. In addition, Chen et al. [20] showed that SRF is a target of miR-133 and that miR-133 overexpression inhibited the *SRF* 3′ UTR luciferase reporter gene. miR-133 overexpression can enhance myoblast proliferation by repressing SRF protein. In contrast, Niu et al. [35] showed a positive correlation between SRF protein and miR-133. In fact, there was a negative feedback loop between miR-133 and SRF protein. SRF controls the muscle-specific expression of miR-133; miR-133 represses SRF expression. We examined whether SRF participates in the protective effects of sEHIs in MI mouse hearts, and observed that the sEHI t-AUCB repressed *SRF* mRNA levels dose-dependently while upregulating SRF protein. It is therefore expected that t-AUCB can restore the impaired SRF protein after ischemia by suppressing miR-133 levels.

### Study limitations

We not examine the relationship between the incidence of ischemic arrhythmia and miR-133 levels in the ischemic myocardium. This was because we were not able to record continuous ECG for longer periods in the mice due to the lack of an implantable telemetry system. Therefore, we focused on investigating the effect of sEHIs on the expression of arrhythmia-related genes. Second, we did not explore whether Ca^2+^ cycling was also involved in this process. In hyperglycemic rats, sEHIs prevented Ca^2+^ deregulation and sarco(endo)plasmic reticulum Ca^2+^-ATPase (SERCA) remodeling [36]. Moreover, miR-133 regulates the proteins involved in Ca^2+^ handling [37]. Therefore, further studies are warranted to investigate whether the anti-arrhythmic effects of sEHIs are also related to Ca^2+^ cycling in the ischemic myocardium.

## Conclusions

In conclusion, the sEHI t-AUCB increases *KCNQ1* and *KCNH2* mRNA and protein by suppressing miR-133 under ischemic arrhythmia conditions. SRF protein upregulation might be a mechanism by which sEHIs reduce miR-133 expression.

## Additional file

Additional file 1: **Figure S1.** Effects of different doses of agomir-133 (15, 25, 40 nM) on expression of miR-133 in ischemic myocardium. **Figure S2.** MicroRNA profile changes between sham and MI mice treated with or without t-AUCB. **Figure S3.** Effect of t-AUCB on primary neonatal mouse ventricular myocytes viability. **Table S1.** Significantly up-regulated miRNAs in ischemic myocardium between sham-operated animals with MI mice treated with or without t-AUCB. **Table S2.** Significantly down-regulated miRNAs in ischemic myocardium between sham-operated animals with MI mice treated with or without t-AUCB. (DOCX 1178 kb)

## Abbreviations

3′ UTR: 3′ untranslated regions
Cx43: connexin 43
DHETs: dihydroxyeicosatrienoic acids
EETs: Epoxyeicosatrienoic acids
ERG: ether-a-go-go related gene
*GJA1*: gap junction protein alpha 1
I_kr_: delayed rectifier K^+^ current
I_Ks_: slow delayed rectifier K^+^ current
*KCNH2*: potassium voltage-gated channel subfamily H member 2
*KCNJ2*: potassium voltage-gated channel subfamily J member 2
*KCNQ1*: potassium voltage-gated channel subfamily Q member 1
MI: myocardial infarction
miR-133: microRNA-133
NF-Κb: nuclear factor Κb
PI3K: phosphatidylinositol 3-kinase
sEHIs: soluble epoxide hydrolase inhibitors
SERCA: sarco(endo)plasmic reticulum Ca2 + -ATPase
*SRF*: serum response factor;
t-AUCB: trans-4-[4-(3-adamantan-1-yl-Ureido)-cyclohe-xyloxy]-benzoic acid
TTC: 2,3,5-triphenyltetrazolium chloride
