# Supplementary material for: Soluble epoxide hydrolase inhibitors, t-AUCB, downregulated miR-133 in a mouse model of myocardial infarction
Source: Lipids Health Dis. 2018 May 29;17:129. doi: 10.1186/s12944-018-0780-y (PMC5975509; doi:10.1186/s12944-018-0780-y)
Supplement: Supplementary file 1 — Figure S1. Effects of different doses of agomir-133 (15, 25, 40 nM) on expression of miR-133 in ischemic myocardium. Figure S2. MicroRNA profile changes between sham and MI mice treated with or without t-AUCB. Figure S3. Effect of t-AUCB on primary neonatal mouse ventricular myocytes viability. Table S1. Significantly up-regulated miRNAs in ischemic myocardium between sham-operated animals with MI mice treated with or without t-AUCB. Table S2. Significantly down-regulated miRNAs in ischemic myocardium between sham-operated animals with MI mice treated with or without t-AUCB. (DOCX 1178 kb) [file 12944_2018_780_MOESM1_ESM.docx]

Soluble epoxide hydrolase inhibitors, t-AUCB, downregulated miR-133 in a mouse model of myocardial infarction

Ya-jun Gui^1^, Da-Li^2^, Jing-yuan Chen^1^, Ya-ting Wang^1^, Jia-hui Hu^1^, Cai-xiu Liao^2^, Li-Min Deng^3^, Qun-yan Xiang^1^, Tao Yang^4^, Xiao Du^1^, Shi-lan Zhang^1^, Dan-yan Xu^1,*^

^1^Department of Cardiovascular Medicine, The Second Xiangya Hospital, Central South University, 139 Middle Renmin Road,Changsha, Hunan 410011, China

^2^Department of Geratology, Internal Medicine, the Third Hospital of Changsha, Changsha, Hunan 410011, China

^3^ Center for Pulmonary Vascular Disease, FuWai Hospital & Cardiovascular Institute Chinese Academy of Medical Sciences & Peking Union Medical College, Beijing, China

^4^Department of Cardiology, Internal Medicine, Changsha Central Hospital, Changsha, Hunan 410011, China

^*^**Corresponding author**: Fax: +8673185295407. E-mail address: xudanyan02@csu.edu.cn (D. Xu)

**Supplemental Material**

**Supplementary Methods**

***Ventricular myocyte isolation and in vitro transfection***

Isolation and culture of neonatal mouse ventricular myocytes was peformed as described in our previsous study [18]. Briefly, primary neonatal mouse ventricular myocytes were harvested from ventricles of 1 day old neonatal Kunming mice. All mice ventricular myocytes were minced, and then digested with trypsin, and the supernatants were collected. Pelleted cells were centrifuged at 1000 rpm for 10 min in a tabletop centrifuge. The cells were then cultured for 24h.

The cells were divided into four groups as follows: (i) control, (ii) agomir-NC: myocytes were transfected with 200 nM agomir-NC for 60 h, (iii) agomir-133: transfected with 200 nM agomir-133 for 60 h, (iv) 20 μM t-AUCB: cells were treated with t-AUCB for 60h, (v) agomir-133+20μM t-AUCB: cells were transfected with 200 nM agomir-133 for 24h and then treated with 20μM t-AUCB for 36h.

***miRNA microarray analysis***

miRNA expression was profiled using a CapitalBio miRNA microarray (Beijing, China). Raw data were normalized and analyzed using Significance Analysis of Microarrays (SAM, Stanford University, Stanford, CA, USA).

***Cytotoxicity of t-AUCB and agomir-133***

The effect of t-AUCB and agomir-133 on neonatal mouse ventricular myocytes viability was determined by measuring the level of LDH activity as described previously [38].

**Figure S1.**

**
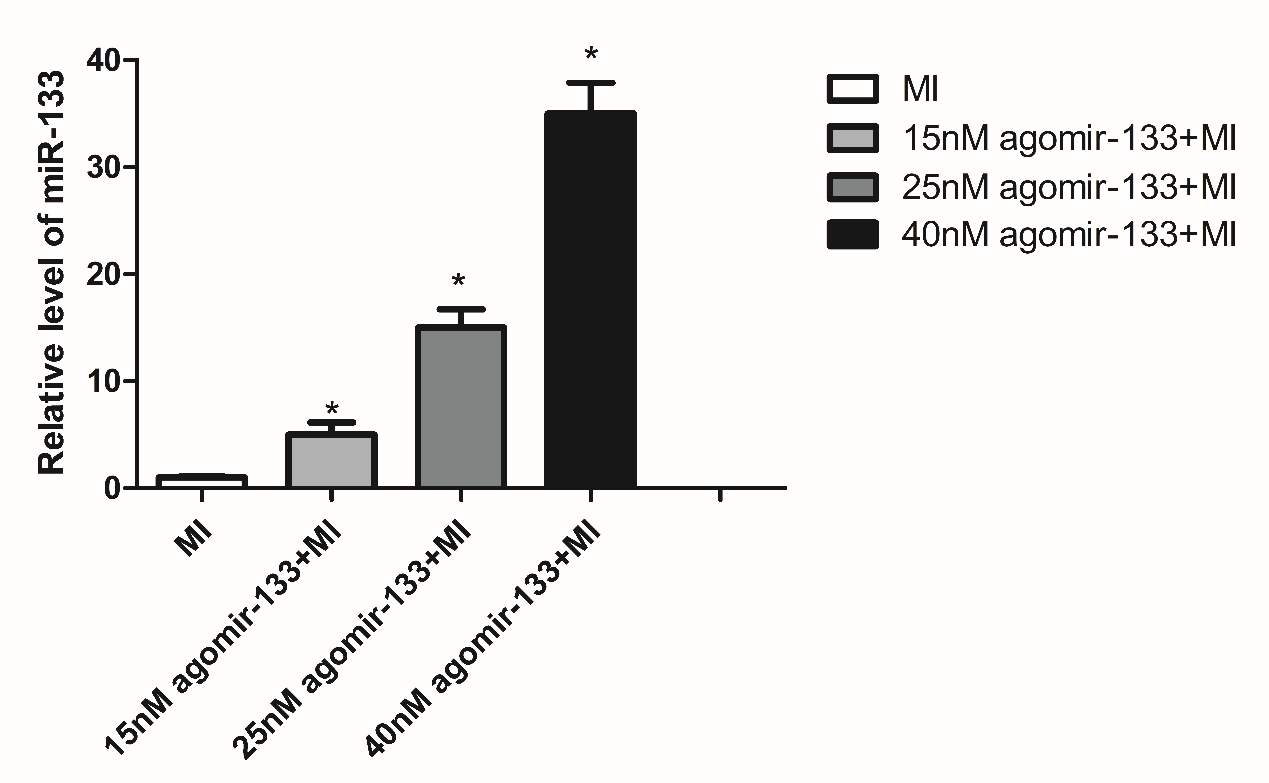
**

**Figure S1. Effects of different doses of agomir-133 (15, 25, 40 nM) on expression of miR-133 in ischemic myocardium.** The mice were injected with PBS or miR-133 agomir (15，25，40 nM) via the tail vein after occlusion. Measurements were made 24h after MI. miR-133 levels were quantificated by real-time PCR. Data were expressed as mean ±SEM; **P* ＜0.05 vs. MI group.

**Figure S2**

**
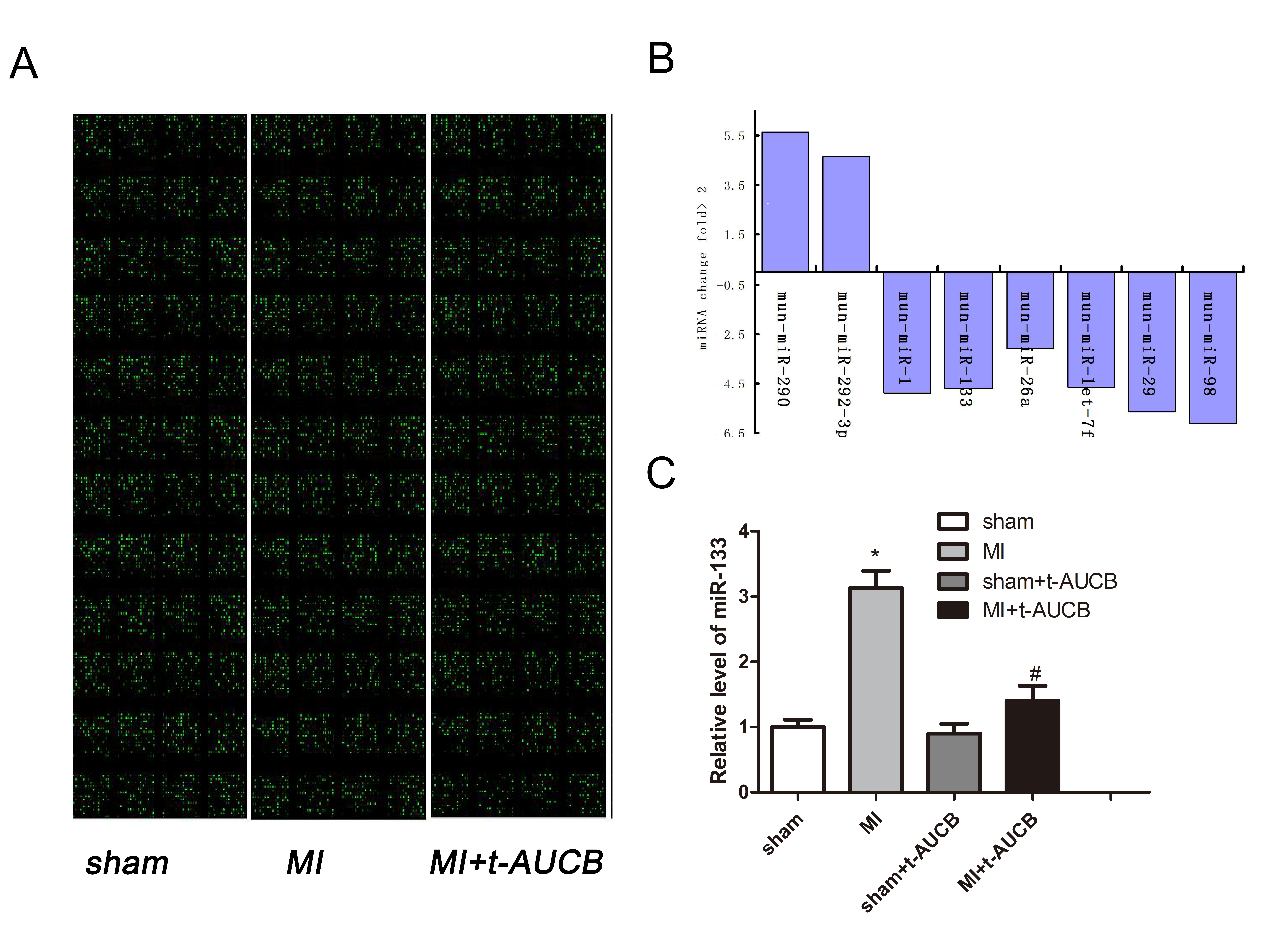
**

**Figure S2. MicroRNA profile changes between sham and MI mice treated with or without t-AUCB.** A. The miRNA microarray results for sham and MI mice treated with or without t-AUCB. B. Bargraph representation of miRs regulated 2 or more fold in ischemic myocardium of MI mice after treated with t-AUCB for 7 days as determined by microarray anlysis. Values are expressed as fold change compared with MI mice. C. Real-time PCR analysis confirmed the regulation of miR-133 in MI mice treated with t-AUCB compared with MI mice. **P*＜0.05 vs. sham group；#*P*＜0.05 vs. MI group; n=3.

**Figure S3.**

**
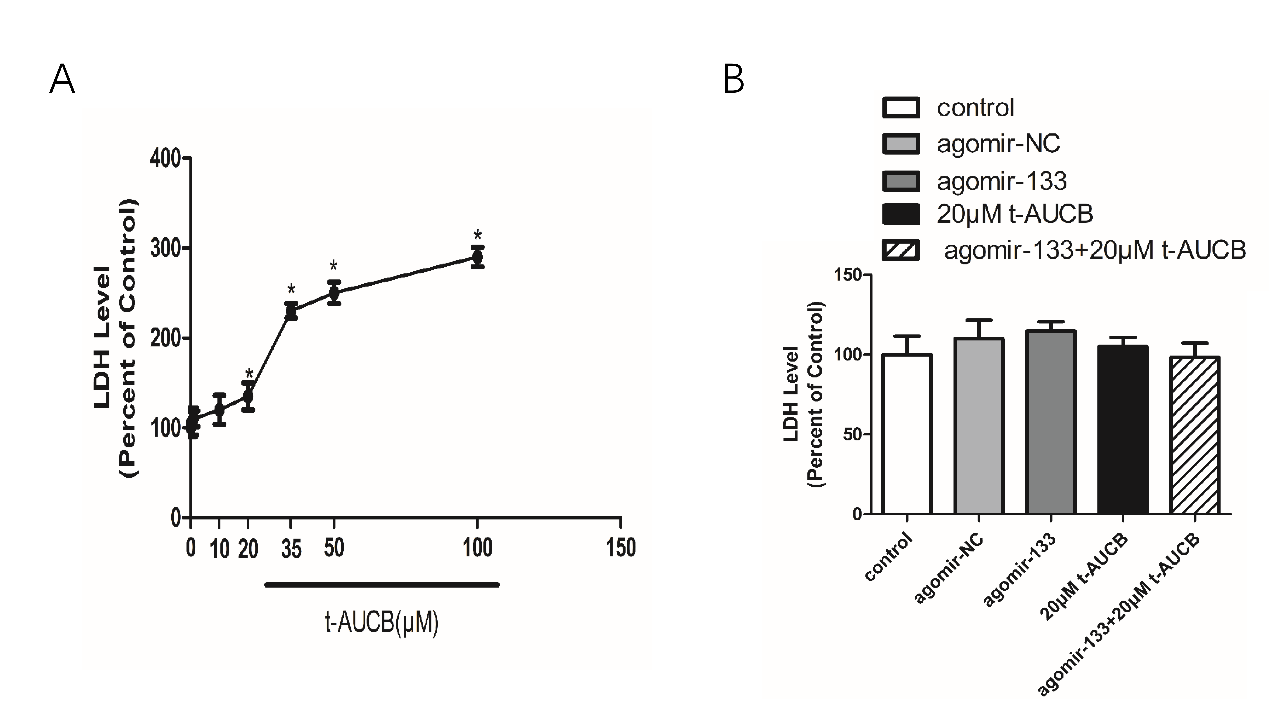
**

**Figure S3. Effect of t-AUCB on primary neonatal mouse ventricular myocytes viability.** A. neonatal mouse ventricular myocytes cells were treated for 4 h with various concentrations of t-AUCB (0, 0.1,0.5,1,10,20,35,50,100 μM). After 24h, cells viability was determined using LDH assay. B. neonatal mouse ventricular myocytes cells were exposed for 4 h to 20 μM t-AUCB in the presence and absence of 200 nM agomiR-133. After 24 h, cells viability was determined using LDH assay. Data were expressed as mean ± SEM; **P*＜0.05 vs. control;

**Table S1.** **Significantly up-regulated miRNAs in ischemic myocardium between sham-operated animals with MI mice treated with or without t-AUCB**


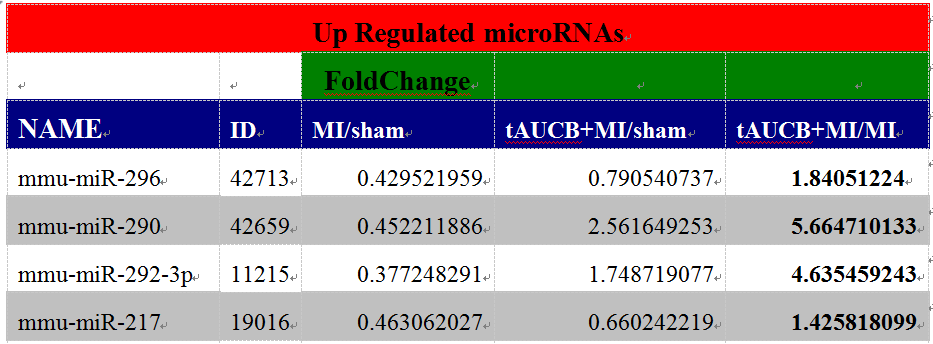


**Table S2. Significantly down-regulated miRNAs in ischemic myocardium between sham-operated animals with MI mice treated with or without t-AUCB**


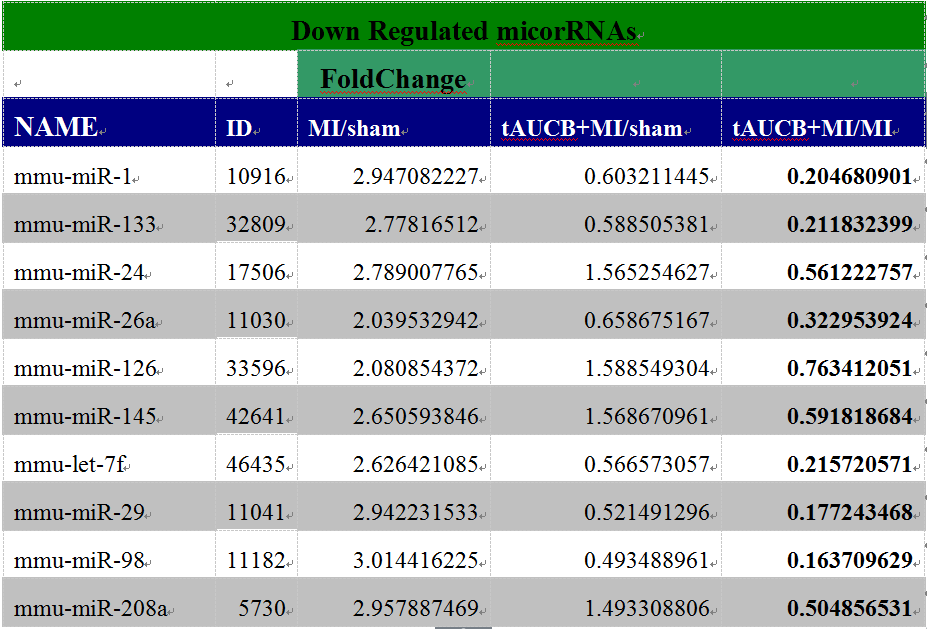


**Reference**

18. Liu Q, Zhao X, Peng R, Wang M, Zhao W, Gui YJ, Liao CX, Xu DY: **Soluble epoxide hydrolase inhibitors might prevent ischemic arrhythmias via microRNA-1 repression in primary neonatal mouse ventricular myocytes.** *Mol Biosyst* 2017,**13**:556-564

38. Maayah ZH, Abdelhamid G, Elshenawy OH, El-Sherbeni AA, Althurwi HN, McGinn E, et al :

**The Role of Soluble Epoxide Hydrolase Enzyme on Daunorubicin-Mediated**

**Cardiotoxicity**. *Cardiovasc Toxicol* 2017*.*
